# Benchmarking the healthiness, equity and environmental sustainability of university food environments in Australia, 2021/22

**DOI:** 10.1186/s40795-025-01029-x

**Published:** 2025-02-11

**Authors:** Gary Sacks, Jasmine Chan, Davina Mann, Sarah Dickie, Alexa Gaucher-Holm, Shaan Naughton, Oriana Ruffini, Ella Robinson

**Affiliations:** 1 Global Centre for Preventive Health and Nutrition, Institute for Health Transformation, Deakin University, Geelong, Australia; 2 Department of Nutrition, Dietetics and Food, Faculty of Medicine, Nursing and Health Sciences, Monash University, Melbourne, Australia; 3 School of Nutrition, Institute of Nutrition and Functional Foods, Université Laval, Québec City, Canada

**Keywords:** University, Food environment, Benchmarking, Population health, Diet, Food retail

## Abstract

**Background:**

Food environments on university campuses have an important influence on the diets of staff and students. This study aimed to assess the healthiness, equitability and environmental sustainability of Australian university food environments, and identify priority recommendations for policy and practice.

**Methods:**

We applied the previously developed ‘Uni-Food’ tool in nine universities (17 campuses, 165 food retail outlets) in Australia between 2021 and 2022. Data on three components: (1) ‘university systems and governance’; (2) ‘campus facilities and environment’; and (3) ‘food retail outlets’ were collected from desk-based policy audits and in-person campus audits. Universities were given an overall score from 0-100, based on their performance across all components.

**Results:**

University scores ranged from 27/100 to 66/100 (median = 46). Universities scored highest in the ‘campus facilities and environment’ component, reflecting that the broad campus environment (including areas such as catering, advertising on campus, and food-related environmental sustainability initiatives) has been an area of focus. Universities scored lowest in the ‘university systems and governance’ component, reflecting a relative lack of policy action, funding and governance in this area, with few initiatives to promote the availability and affordability of healthy and environmentally sustainable foods.

**Conclusion:**

Stronger action is needed to improve Australian university food environments, including in food retail outlets, vending, catering and at campus events. Universities can demonstrate leadership by implementing university-wide policies that limit the availability of unhealthy foods and beverages (e.g. sugary drinks) on campus, and setting targets for the proportion of healthy and environmentally sustainable foods procured and sold on campus. Other stakeholders, including governments, can play a role in incentivising universities to adopt recommended actions.

**Supplementary Information:**

The online version contains supplementary material available at 10.1186/s40795-025-01029-x.

## Background

### Introduction

Food environments are the interface between the food supply chain and consumers [1]. Food environments include the surroundings, opportunities and conditions that influence people’s food and beverage choices and nutritional status [2]. Food environments across the globe are becoming increasingly unhealthy, inequitable and unsustainable, dominated by unhealthy ultra-processed foods and beverages that are heavily promoted, with decreased availability and affordability of nutritious minimally-processed food [3, 4]. These food environment changes are widely recognized as the primary driver of unhealthy and unsustainable diets, which have wide ranging negative impacts on population and planetary health [4, 5]. In Australia, unhealthy diets are the leading contributor to the burden of disease, with lower socio-economic groups experiencing poorer diets and associated health outcomes [6]. Additionally, Australian food production and consumption is responsible for more than a third of national greenhouse gas emissions, as well as substantial biodiversity loss, land clearing, pollution of waterways and food waste [7].

Urgent and comprehensive action across multiple settings is needed to improve the healthiness (consistent with relevant dietary guidelines), equity (including access to, and affordability of, culturally diverse healthy and environmentally sustainable foods and beverages), and environmental sustainability (with a focus on the environmental impact of food sold and consumed) of food environments. Universities represent an important setting for action, due to the strong influence that university food environments have over the diets of students and staff, and the potential leadership role that universities can play in demonstrating best practice for society more broadly [8, 9]. In Australia, the higher education sector catered to over 1.6 million students and over 100,000 staff in 2021 [10], which equates to almost 7% of the 2021 Australian population, the majority of whom are young adults (18–24 years old). Improving diets amongst young adults is of particular importance considering more than 46% of 18–24 year olds in Australia now live with overweight or obesity (2017), with weight gain in adolescence and young adulthood substantially increasing the risk of weight gain later in life and preventable chronic health conditions (e.g., heart disease, type 2 diabetes, various cancers) [11]. Actions and interventions to improve university food environments are an important avenue for driving positive dietary changes amongst young adults in Australia and helping establish healthier dietary behaviours into adulthood.

There have been several previous studies that have examined the healthiness of university food environments. These studies predominantly assessed the healthiness of food outlets on university campuses, and found that unhealthy foods and beverages were more widely available and promoted than healthy foods and beverages [12–17]. However, there have been no studies that have comprehensively assessed multiple health, equity, and environmental dimensions of university food environments. As such, there is limited understanding of the extent to which university policies (documented strategies or statements of principles that regulate university operations) and practices (usual actions and procedures that occur at the university) promote or inhibit healthy, equitable and environmentally sustainable staff and student diets, and the potential opportunities for improving the ways in which foods and beverages are supplied, promoted, priced, and distributed on university campuses.

### The Uni-Food tool

In light of this evidence gap, in 2020, INFORMAS (the International Network for Food and Obesity / Non-communicable Diseases (NCDs) Research, Monitoring and Action Support) developed the University Food Environment Assessment (Uni-Food) tool to monitor and assess the healthiness, equitability and environmental sustainability of university food environments [18]. INFORMAS is a global network of researchers that aims to monitor, benchmark and support public and private sector actions to increase healthy food environments [2]. The process for developing and implementing the tool was adapted from INFORMAS methods for assessing the public sector and private sector on their policies and actions related to food environments [19, 20]. Developed in conjunction with an expert working group of research and professional staff from eight Australian universities, the tool was created based on global best practice recommendations for the creation of healthy and environmentally sustainable food environments at universities [18]. The tool was designed to assess the extent of policy and practice implementation at a particular university, taking into account attributes such as comprehensiveness, quality and reach.

The Uni-Food tool consists of three main components, divided into 16 domains, and 61 indicators (refer to Table 1 for overview), with the components weighted according to their level of importance [18]. The ‘university systems and governance’ component evaluates: university leadership and planning; formal university strategies and policies; monitoring and reporting on key food environment characteristics; funding and resources allocated to support food environment activities; and engagement of staff and students in the design of food environments. The ‘campus facilities and environment’ component assesses physical and social environments on campus, including programs and facilities that: promote the availability, accessibility, and affordability of healthy, equitable and environmentally sustainable food; reduce promotion of unhealthy and unsustainable food; improve student and staff skills and knowledge around healthy and environmentally sustainable food; and reduce the environmental impact of food sold and consumed on campus. The ‘food retail outlets’ component relates to food retail stores on university campuses, regarding the availability, accessibility, promotion, price, and environmental impact of food sold, and information provided at point of sale.

**Table 1:** Overview of components, domains, sub-domains, indicators and weightings of the University Food Environment Assessment tool, from Mann et al. [18]

Component	Component weighting	Domain	Sub-domain (number of indicators, *n*)	Domain weighting
**University systems and governance**	**40%**	Leadership and planning	Policies and commitments (*n* = 2)	10%
		Policies for food retail environments	Proportion of healthy and environmentally sustainable food and beverages sold (*n* = 1)	50%
			Restrictions on availability (*n* = 1)	
			Food pricing (*n* = 1)	
			Labelling and information (*n* = 2)	
			Food retail contracts (*n* = 1)	
		Monitoring and reporting	Food environments (*n* = 1)	10%
			Staff and student population (*n* = 2)	
		Funding and resources	Funding (*n* = 1)	20%
			Resources (*n* = 2)	
		Stakeholder engagement	Platforms for interaction (*n* = 1)	10%
			Student voice (*n* = 3)	
**Campus facilities and environment**	**40%**	Availability and accessibility	Drinking water (*n* = 1)	20%
			Healthy, equitable and environmentally sustainable food (*n* = 1)	
			Culturally appropriate food (*n* = 2)	
			Vending machines (*n* = 3)	
			Self-catering facilities (*n* = 1)	
			Operating hours (*n* = 1)	
		Equity	Food affordability (*n* = 2)	25%
			Food relief (*n* = 1)	
		Advertising and sponsorship	Advertising (*n* = 1)	10%
			Sponsorship (*n* = 1)	
		Catering and events	Catering (*n* = 1)	15%
			Fundraising (*n* = 1)	
			Student accommodation (*n* = 1)	
		Personal and community development	Community skills building (*n* = 3)	15%
			Training and information (*n* = 3)	
		Environmental impact	Waste and recycling (*n* = 3)	15%
			Food packaging and serving ware (*n* = 1)	
			Water (*n* = 1)	
			Energy and emissions (*n* = 1)	
**Food retail outlets**	**20%**	Availability and accessibility	Healthy, equitable and environmentally sustainable foods and beverages (*n* = 2)	25%
			Portion sizes (*n* = 1)	
			Location of foods (*n* = 1)	
		Promotion	Food and beverage advertising (*n* = 1)	15%
		Price	Relative prices (*n* = 2)	25%
			Price promotions (*n* = 2)	
		Information	Nutrition information (*n* = 1)	15%
			Cultural information (*n* = 1)	
			Environmental sustainability information (*n* = 1)	
		Environmental impact	Food packaging and serving ware (*n* = 2)	20%
			Food waste (*n* = 1)	

For each indicator as part of the Uni-Food tool, a maximum score of 10 points is allocated where university policy and practice implementation aligns with a best practice statement [18]. High scores are given for comprehensive implementation of policies and practices, along with regular monitoring and reporting, across relevant settings (e.g., all campuses). Where implementation falls below best practice, lower scores are assigned. An example of an indicator and scoring criteria are provided in the supplementary material (Figure S1). Indicators are grouped within sub-domains and domains, which are weighted by domain weightings to allocate a component score (expressed as a %) (refer to Table 1). Components are weighted by component weightings to derive an overall score (out of 100) for each university. The overall score for each university is designed to reflect the extent of implementation of best practice (0–20%: weak or no implementation; 20–40%: low implementation; 40–60%: medium implementation, 60–80%: moderately high implementation; 80–100%: strong implementation).

### Study aim

This study reports the results of the implementation of the Uni-Food tool in nine Australian universities in 2021/22. The aim of this study was to assess the healthiness, equitability and environmental sustainability of Australian university food environments using the Uni-Food tool, and identify priority recommendations for policy and practice.

## Methods

### Selection of universities

Convenience sampling was used to recruit universities from across Australia, with the intention of including as many universities as possible, with diversity in both geographic location and campus sizes. Suitable contacts at almost all Australian universities with physical campuses (*n* = 40) were identified through a community of practice that was part of the Nourish Network– a multi-sector collective working collaboratively to transform food retail practices. In 2021, invitations to participate in the assessment were extended via email to the identified contacts. For each university that agreed to participate, university representatives agreed for their universities to be named in the reports and materials that resulted from the research.

### Assessment of university food environments using the Uni-Food tool

**Fig. 1:**
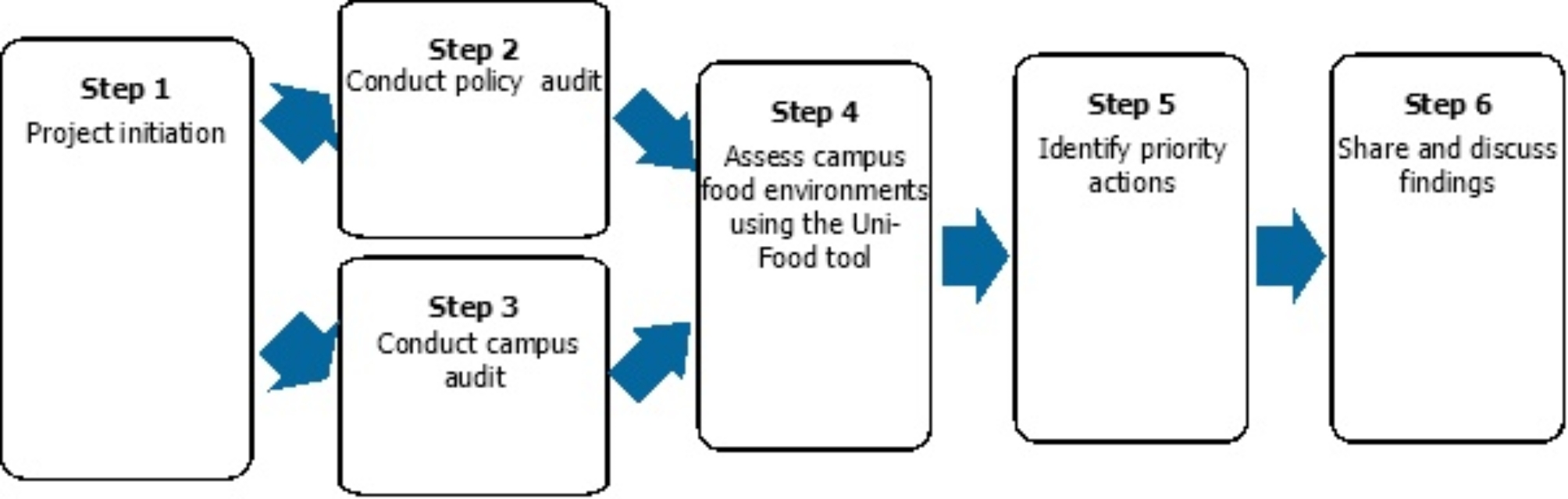
The Uni-Food tool assessment process at each university, based on Mann et al. [18]

The process that we (the research team) used to implement the Uni-Food tool at each university is outlined in Fig. 1 and described in more detail below.

#### Step 1: Project initiation

Universities that had agreed to participate in the assessment process nominated a university project champion to oversee the process. Their role included gaining internal support for the project at the university, establishing implementation timelines, and recruiting assessors to conduct the policy and campus audits. Assessments were generally conducted by students as part of student projects or placements in a related field, i.e., health promotion, nutrition or environmental studies. The research team supported project champions throughout the assessment process.

#### Steps 2 and 3: Conduct university audit (including policy audit and campus audit)

The research team trained nominated assessors at each university in how to conduct the university audit, and they provided support to the assessors throughout the audit process. As part of the policy audit, documented evidence of university policies and actions related to the ‘university systems and governance’ component of the Uni-Food tool were collected by assessors via a search of university websites, intranet and policy databases. Where required, further input was sought from university staff members with knowledge of processes not formally documented or available to the general public, such as those in retail and campus operations roles, supported by the project champion.

For the campus audit, each university team established the scope (i.e., number of campuses and food retail outlets to be included). The campus audit involved assessors visiting physical campuses and taking written notes and photo evidence to assess the ‘campus facilities and environment’ component of the Uni-Food tool, including the presence of food advertising, vending machines, availability of drinking water, recycling and waste management, and community gardens. The food retailer audit involved assessors visiting and taking written notes and photo evidence to assess the ‘food retail outlets’ component of the Uni-Food tool. For this component, food retail outlets were defined as outlets on campus that sold predominately ready-to-eat foods (including university-run cafeterias and cafés). Retailers that sold food or ingredients that required further preparation for consumption (e.g., supermarkets) and those that only sold drinks (e.g., coffee shops/carts) were excluded. ‘Healthy’ foods and beverages were defined based on the Australian Dietary Guidelines and other relevant Australian state and territory government guidelines. ‘Environmentally sustainability’ concepts were focused on reducing the environmental impact of food sold and consumed on campus, including minimising food waste and food packaging, preferencing fresh and minimally-processed food, and limiting red and processed meat [18]. ‘Equity’ concepts were focused on access to, and affordability of, culturally diverse healthy and environmentally sustainable foods and beverages. Further details on the process for classification are available in an earlier paper [18]. Campus audits were predominantly conducted in publicly accessible areas, unless otherwise agreed by the university.

All policy and campus audit data were collected at a single time point for each university, in either 2021 or 2022. Assessors entered data into an online data entry form hosted on REDCap [21].

#### Step 4: Assess campus food environments using the Uni-Food tool

For each university, two to three assessors independently conducted the scoring of indicators, using the REDCap platform [21]. Indicator scoring (out of a maximum of 10 points per indicator) was based on the evidence collected and the scoring criteria of the Uni-Food tool. In an effort to ensure reliability in the scoring across universities, evidence and scoring for each indicator from all universities were downloaded from REDCap into Excel, and cross-checked by a member of the research team (SD), with any discrepancies in scoring discussed amongst multiple members of the research team. Where the research team considered that there was not enough evidence provided for a particular indicator, a member of the research team contacted the relevant assessors to request more information before assigning the final score.

As per the methods for applying the Uni-Food tool [18], for each university, scores in each domain and component were converted into a score out of 100, and universities were given an overall score out of 100, based on their weighted performance across the three components of the tool. Gwet’s AC1 (unweighted) integrated reliability coefficients (calculated using Rstudio) were used to calculate the inter-rater reliability for each university assessment score.

#### Step 5: Identify priority actions

Priority actions for each university were developed by the research team, in collaboration with the project champion at each university. Priority recommendations were developed using an iterative process, taking into account the level of implementation of policies and practices across each domain of the Uni-Food tool, as well as considerations of feasibility, alignment with existing policies and practices, and performance and actions of other universities. The process of development of the priority actions involved a meeting between the research team and the project champion, where they were given an overview of the main results and strengths/areas for improvement. Proposed actions were then refined and prioritized collaboratively via email.

#### Step 6: Share and discuss findings

A summary report was prepared for each university. The report included a scorecard on how the university performed in each domain, highlighting areas of strength and recommendations for action. The report was shared with key staff members at the university, as identified by the project champion.

Following the sharing of individual university reports, an overall report for all universities was also prepared. The overall report included scoring for each university, and benchmarking of performance across universities in each domain and component. Overall areas of strength and recommendations were identified for the university sector.

### Analysis

We collated all scores across the participating universities, and assessed patterns of performance across all the participating universities. We used descriptive statistics to highlight median (as well as interquartile ranges, IQRs) scores bethiy domain and component (expressed as scores out of 100). University scores were grouped according to their level of action/implementation (0–20% = very low; 20–40% = low; 40–60%: Moderate; 60–80% = Moderately high; 80–100% = High). We compiled key areas of strength across universities, as well as key recommendations, to outline overall strengths and recommendations for the university sector in Australia.

## Results

### Universities included in the assessment

A total of nine universities were included in the assessment, located in New South Wales (NSW) (*n* = 4), Victoria (VIC) (*n* = 2), Queensland (QLD) (*n* = 1), Tasmania (TAS) (*n* = 1) and South Australia (SA) (*n* = 1). University student populations ranged between 25,531 and 87,115. A total of 17 campuses and 165 food retail outlets were assessed across universities using the Uni-Food tool (refer to Table 2).

**Table 2:** Characteristics of universities and number of campuses and food retailers assessed

University	State	Year assessed	Number of students^1^	Number of campuses assessed	Number of food retailers assessed
Deakin University	VIC	2022	62,868	2	11
Flinders University	SA	2021	25,531	2	11
Macquarie University	NSW	2022	44,895	1	24
Monash University	VIC	2022	87,115	1	34
University of Queensland	QLD	2022	56,220	3	29
University of Sydney	NSW	2022	77,431	1	32
University of Tasmania	TAS	2022	36,367	3	11
University of Wollongong	NSW	2021	32,000	1	17
Western Sydney University	NSW	2022	48,614	3	9

^1^Australian Government Department of Education, 2023 [22]

### Overall performance

**Fig. 2:**
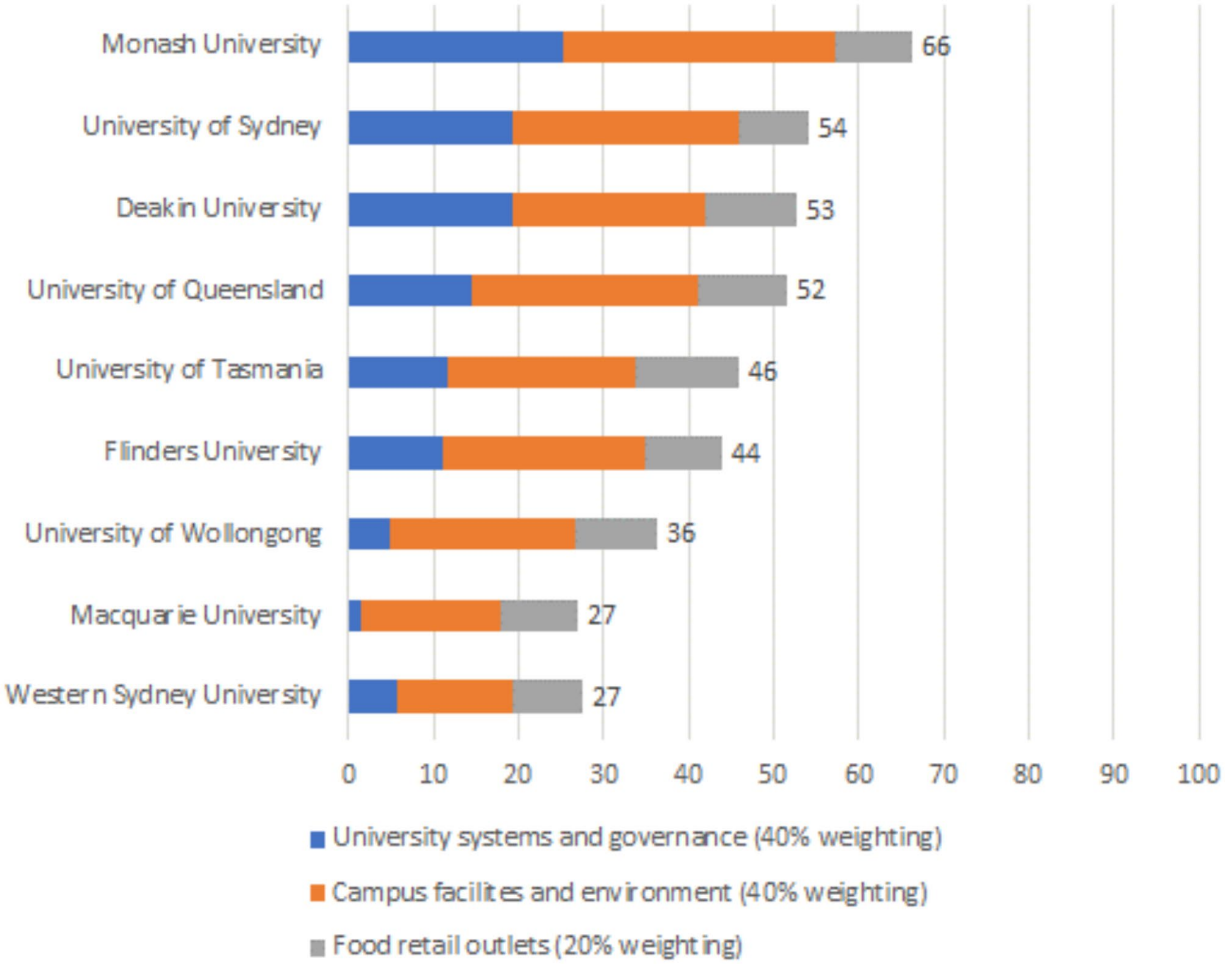
Assessment of the healthiness, equity, and environmental sustainability of food environments at nine Australian universities

University scores ranged from 27/100 to 66/100 (median: 46; IQR: 17). Monash University was the highest scoring university overall (66/100), with Macquarie University and Western Sydney University receiving the lowest overall score (27/100) (refer to Fig. 2). Inter-rater reliability of the scoring for each university using Gwet’s AC1 (unweighted) integrated reliability coefficients was similar across universities (median: 0.72; IQR: 0.03).

### Performance by component

University performance across the domains and components of the tool are provided in the supplementary material (Table S1).

#### University systems and governance

The ‘university systems and governance’ component assessed high-level policies and governance structures that guide university-wide processes and executive-level commitment to improving the health and environmental sustainability of campus food environments. This was the lowest scoring component overall (median: 29; IQR: 34). Leading universities in this component were Monash University (63/100), the University of Sydney (48/100) and Deakin University (48/100). Universities performed best in the ‘leadership and planning’ domain (median: 60; IQR: 25), with six out of nine universities taking at least moderate action (i.e., scoring above 40%) to embed health, equity and environmental sustainability within high-level policies and commitments. Deakin University’s ‘Food Charter’ was a notable good practice example. The Food Charter outlined five guiding principles (healthy, informed, balanced, easy and sustainable) to promote increased affordability and accessibility of healthy and environmentally sustainable food options, availability of nutritional information, and reduced food waste in retail food outlets.

The lowest scoring domain was ‘policies for food retail environments’ (median: 8; IQR: 31), with few examples of comprehensive policies to address the healthiness, equity, and environmental sustainability of food retail environments. We found that ‘monitoring and reporting’ related to food environments was either not conducted at all or fragmented (e.g., not covering comprehensive domains of health, equity and environmental sustainability) and irregular (median: 27; IQR: 25), and there were few universities that had dedicated ‘funding and resources’ or ‘stakeholder engagement’ activities to promote and support better university food environments (median: 53 and 58, respectively). Monash University demonstrated leading practice in several domains within this component. Monash University had developed policies and associated contractual requirements for healthy food and beverage provision, alongside comprehensive monitoring of campus food environments, with dedicated staff to support the development, implementation and monitoring of initiatives related to food environments.

#### Campus facilities and environment

The ‘campus facilities and environment’ component assessed campus facilities and the extent to which campus environments encourage healthy and environmentally sustainable food choices and promote the health and wellbeing of staff and students. This was the highest scoring component overall (median: 57; IQR: 12). Universities scored highest in the ‘environmental impact’ domain (median: 73; IQR:10), with all universities having taken some action to address food-related environmental impact concerns across campuses, for example through diverting food waste from landfill. Universities also scored well in the ‘personal and community development’ domain (median: 69; IQR: 31), with most universities having initiatives in place to develop skills and awareness around healthy and environmentally sustainable food (e.g., through community gardens). Although universities scored moderately high in the ‘advertising and sponsorship’ domain (median: 65; IQR: 15), only the University of Tasmania was largely free from unhealthy advertising and sponsorship across campuses. Seven out of nine universities had taken at least moderate action (i.e., scoring above 40%) in the ‘equity’ domain (median: 57; IQR: 13), for example through food relief programs. Only two universities (The University of Sydney, Monash University) had taken strong action (i.e., scoring above 80%) to improve the health and environmental sustainability of ‘events and catering’, which was the lowest scoring domain in this component (median: 38; IQR: 40). In this domain, the University of Sydney’s ‘Healthy Choices Catering Guidelines’ was notable for providing comprehensive evidence-based recommendations for serving healthy food and beverages at events. Monash University practice was notable in the ‘availability and accessibility domain’ for their implementation of a healthy vending procedure whereby vending machines across all campuses contain competitively priced, prominently-placed healthier snack and beverage choices that were labelled with ‘traffic light’ labelling (designed to indicate the healthiness of foods).

#### Food retail outlets

The ‘food retail outlets’ component assessed the characteristics of food retail outlets on university campuses. The median score for this component was 46 (IQR: 7). University food retailers generally scored well in the ‘promotion’ domain (median: 72; IQR: 14), with the audits showing that almost half (49%, data available on request) of food retail outlets across universities surveyed had no or very few advertisements for unhealthy foods and beverages. All food retail outlets had a combination of unhealthy and healthy food options available to purchase, although unhealthy options dominated, with universities receiving moderate to low scores in the ‘availability and accessibility’ domain (median: 45; IQR: 10).

In the ‘pricing’ domain (median: 56; IQR: 8), we found that the vast majority (69%, data available on request) of food retail outlets surveyed did not have price promotions that would encourage the purchasing of larger portion sizes; however, pricing arrangements across outlets generally did not incentivize the purchase of healthy over unhealthy or vegetarian over meat-containing options. Four universities (Deakin University, The University of Tasmania, The University of Queensland, University of Wollongong) had food retail outlets that were taking moderate action (i.e., scoring above 40%) in the ‘environmental impact’ domain (median: 39; IQR: 16) to ensure food packaging and serving ware was reusable, and had some food waste reduction programs in place. Nevertheless, we found widespread use of single-use packaging in food retail outlets.

The lowest scoring domain in this component was ‘information’ (median: 25; IQR: 10), with no universities considered to be providing comprehensive nutrition, environmental sustainability and cultural (e.g. halal) information on food options.

### Priority actions for universities

A set of recommendations was developed for the university sector, by domain and component, based on the results for each university and the overall results (refer to Table 3).

**Table 3:** Recommendations for universities to improve the healthiness, equity, and environmental sustainability of campus food environments

Component and domain	Recommendation
**University systems and governance**	
Leadership and planning	Embed health, equity and environmental sustainability considerations related to food environments within high level university strategies and policies. As part of such strategies and policies, include a publicly available, comprehensive plan outlining measurable and time-bound goals and objectives to promote healthy, equitable and environmentally sustainable food environments. Include elements related to healthiness, cultural diversity, affordability, and environmental sustainability of foods available on campus, and the way in which they are promoted.
Policies for food retail environments	Create university-wide policies that limit the availability and promotion of unhealthy foods and beverages (e.g. sugary drinks) on campus, including in food retail outlets and vending machines. As part of this policy, set SMART (Specific, Measurable, Attainable, Relevant, Timebound) targets for the proportion of healthy and environmentally sustainable foods procured, provided and sold on campus.
Monitoring and reporting	Regularly monitor the healthiness, equity, and environmental sustainability of food environments on campus, including products available, retail environments, campus services, and staff/student diets and food security.
Funding and resources	Allocate funding and resources to research, projects and systems that promote healthy, equitable and environmentally sustainable food environments (e.g. dedicated staffing).
Stakeholder engagement	Ensure students are represented on key working groups responsible for promoting healthy, equitable and environmentally sustainable campus food environments.
**Campus facilities and environment**	
Availability and accessibility	Develop policies and associated contractual arrangements that promote the sale and consumption of healthy food and beverages (in alignment with relevant government dietary or nutrition guidelines) from vending machines, through increased availability and prominent placement of healthier items, and increased nutrition information signage.
Equity	Provide support to retailers to provide healthy, equitable and environmentally sustainable food that is affordable and commercially viable.
Advertising and sponsorship	Create university wide policies restricting the placement and promotion of unhealthy foods and beverages (and associated brands) on university campuses, including sponsorship of events.
Events and catering	Introduce policies and processes to promote the provision of healthy and environmentally sustainable foods and beverages as part of catering and catered events.
Personal and community development	Ensure there is a campus community garden with produce readily accessible to staff and students, and associated programs to build skills and knowledge related to healthy and environmentally sustainable food.
Environmental impact	Introduce comprehensive formal waste monitoring and reduction programs, and implement a policy to support the coordination of food redistribution across the university.
**Food retail outlets**	
Availability and accessibility	Introduce a policy on the provision of predominantly healthy and environmentally sustainable foods and beverages at food retail outlets. As part of this policy, implement restrictions on the placement of unhealthy foods and beverages in prominent locations within outlets.
Promotion	Introduce a policy to restrict the advertising of unhealthy foods and beverages (and associated brands) within food retail outlets across the university, and audit retailers on a regular basis to ensure compliance.
Price	Restrict the use of price promotions and meal deals that encourage the purchase and consumption of unhealthy food and beverages at food retail outlets.
Information	Ensure all campus food retailers provide interpretive nutrition information (e.g., traffic light labelling) and information on dietary requirements (e.g. vegan, gluten free, halal) for all food and beverages sold.
Environmental impact	Support campus food retailers to implement comprehensive waste monitoring and reduction programs, and introduce mandatory policies on the use of reusable/recyclable/biodegradable packaging and serving ware across all outlets.

## Discussion

This study of nine universities in Australia found a lack of comprehensive action to address the health, equity, and environmental sustainability of campus food environments. While the study identified some examples of good practice, overall university scores were relatively low (median score: 46/100; range: 27/100 to 66/100), with substantial diversity of policy and practice, and substantial room for improvement across the board. The study identified a set of concrete priority recommendations for the university sector to improve food environments.

A key finding from this assessment was that almost all universities lacked comprehensive high-level policies and commitments that would address multiple dimensions of food environments (health, equity and environmental sustainability). We found that universities generally had stronger policies for addressing environmental sustainability compared with health and equity concerns, usually as part of university-wide sustainability strategies. This is in line with findings from other studies that have found relatively high prevalence of plans for sustainability at university campuses in high-income countries [8, 9, 23]. The focus on environmental sustainability is potentially a product of decades of international declarations and advocacy efforts to address sustainability in higher education settings [23]. We found no examples of university sustainability strategies that included health or equity considerations related to food environments. The absence of institutionalized policies to address multiple dimensions of food environments, and associated SMART (specific, measurable, achievable, realistic, timebound) targets and reporting of university progress in this area, is likely to lead to sporadic and poorly enforced approaches to improving campus food environments. Future research that explores the drivers of change to university-wide sustainability strategies is warranted.

Our findings from campus audits revealed that campus food retail outlets were generally dominated by unhealthy food options, with widespread use of single-use packaging, limited use of interpretive, easy-to-understand labelling, and a lack of pricing arrangements that would encourage healthy and environmentally sustainable purchases at an affordable price. These findings are similar to previous assessments of university food environments in Australia and globally, which have shown that food retail outlets at university campuses generally promote unhealthy foods and beverages, with limited accessibility and affordability of healthy options [13, 14, 16]. We also note that the wide diversity of areas of assessment, as well as methods and tools used as part of previous studies limits the ability to make detailed comparisons of results [24].

The findings from this study are of particular concern considering that universities are key settings that influence young people’s food access and food choices. Recent data from Australia has found that young people (aged 19–30 years) are some of the highest consumers of unhealthy foods [25], with a recent study also showing that almost 45% of their dietary energy intake came from ultra-processed foods and beverages [26]. Moreover, systematic review evidence of food insecurity in Australia has found that university students experience higher levels of food insecurity in comparison to the general population in Australia [27, 28]. There is a clear role for universities to play in supporting healthy and environmentally sustainable diets amongst young people, including ensuring that healthy and environmentally sustainable food options are affordable and readily accessible across the campus environment [29].

The lack of comprehensive policies and practices to support healthy, equitable and environmentally sustainable food environments across Australian universities in this assessment points to the importance of increased advocacy and accountability for action in the sector. Benchmarking is used broadly within the university sector as a tool for driving performance. With respect to food environments, monitoring and benchmarking of progress against best practice is increasingly recognized as an important mechanism in strengthening accountability efforts, and has been widely applied with respect to both the public and private sectors [30]. There is substantial potential for the Uni-Food tool to be applied more broadly both in Australia and internationally, including as part of INFORMAS that is active in over 65 countries [31]. Widespread application of the tool would facilitate increased benchmarking of performance, including across countries and over time, as well as identification of best practice. The Uni-Food tool could also be used to inform and supplement existing tools that assess university practices. One example is the Sustainability Tracking, Assessment & Rating System (STARS), which is a self-reporting framework, predominantly applied in the United States context, for universities to measure their sustainability performance [32]. STARS recognises sustainability progress and performance by universities (via a tiered rating system) to encourage institutions to improve their rating. STARS includes some indicators related to the environmental sustainability of food environments (e.g., through prioritising the purchase of plant-based and sustainably produced food and beverage items). The Uni-Food tool could be used to expand the STARS assessment of university sustainability across multiple dimensions of food environments.

The results of the assessments conducted as part of this study, and use of the Uni-Food tool more generally, can be used as part of advocacy for improving the health, equity and environmental sustainability of university food environments. All of the universities included in this assessment have health and environmental departments, with research groups dedicated to addressing food-related population and planetary health impacts. Many of the project champions that were involved in this study were working in these departments. Staff and students within these departments could play a key role in advocating for their respective universities to adopt stronger policies on healthy, equitable and environmentally sustainable food environments. Indeed, a number of the project champions from this study indicated that the Uni-Food tool and associated reports [18] were useful for advocating for change within their university. There is growing concern and advocacy around food security of students and young people attending university [17, 27, 33–35], which is likely to provide avenues for raising the profile of university food environments as a key intervention point for improving the health and wellbeing of students.

Australian governments currently have policy guidelines for healthy food provision in certain public sector settings, such as public health services, public schools and local government facilities. In Victoria, for example, the Healthy Choices guidelines include mandatory requirements for public health services which stipulate that all vending machines and in-house managed retail food outlets must have at least 50 per cent ‘healthy’ food (i.e., ‘core’ foods according to the Australian Dietary Guidelines) and no more than 20 per cent ‘unhealthy’ food (i.e., ‘discretionary’ foods), with no sale or promotion of sugary drinks [36]. Similarly, the National Healthy School Canteen guidelines recommend that discretionary foods and drinks are not made available at canteens, and each state and territory has specific mandatory requirements for public school canteens [37]. Whilst private sector health services, workplaces, sports and recreation settings and schools are encouraged to adopt relevant guidelines, there is no requirement or consistent monitoring of food provision in these settings. The vast majority of universities in Australia are public sector institutions, with complex funding arrangements that include government research and teaching grants, local and international student fees, state government funding and investment income [38]. Despite universities being classified as public sector institutions, there are currently no government requirements for these settings to promote healthy, equitable and environmentally sustainable diets. In light of the findings from this study, governments in Australia could consider expanding healthy food provision mandates (currently in force at public health services) to the university sector, and providing increased incentives and recognition for university action in this area. Ideally, guidelines would be nationally consistent.

This study has several strengths. First, the study provided the most comprehensive picture of the current state of food environments at universities in Australia, including assessment of policies, campus environments and food retail outlets. Previous assessments of university food environments have typically only assessed food retail outlets, thus potentially missing other key settings and factors that influence staff and student diets [12, 14, 16, 17]. Second, the findings provide a novel contribution to the literature on complex food environments in Australia, through in-depth assessment of university food environments across multiple dimensions of health, equity, and environmental sustainability. Previous tools assessing food environments in place-based settings have been limited to health criteria only. Given the intersection between the drivers of unhealthy diets, inequities in diet-related health outcomes, and environmental sustainability, and the significant potential for triple duty actions that address health, equity and sustainability [4], the Uni-Food tool methodology provides an important framework for future food environment monitoring and assessment tools designed to measure complex food environments. Future research should explore the transferability of the Uni-Food tool to other settings, such as food courts and health-care settings.

A limitation of the data collection and assessment process was that different assessors, with varying background experience and expertise related to food environments, were used at each university which may have influenced the consistency with which the tool was applied. We aimed to mitigate discrepancies in the assessment by providing consistent training, support and tools to assessors, and by reviewing and cross-checking the data and scoring. While we found strong and consistent inter-rater reliability, we note that future assessments should aim to ensure maximum consistency in the assessment approach.

The study included only nine of the 41 universities in Australia, with the selection of universities for inclusion in the study driven mostly by self-expressed willingness to participate. Due to self-selection bias, the overall average scores from this study may not be representative of the sector overall, with the scores from universities in this study likely to be higher than others as their self-selection likely indicates greater interest in food environment issues. Despite the ‘opt-in’ nature of participation, the study nevertheless included good geographic diversity, and the results showed a range of performance (range of overall scores: 27/100 to 66/100). As part of the process of implementation, we allowed universities the flexibility to determine the timing of the assessment, and the campuses and number of food retail outlets to be included in the scope of assessment. This flexibility was provided to maximise participation and increase feasibility of data collection, particularly in consideration that we did not provide funding to support each university with data collection activities. While each university included their largest campus (by student numbers) in the assessment and multiple food retail outlets, it is possible that the assessment may not have been representative of food environments across the university. Future research using the Uni-Food tool should aim to include all universities in a particular region, and all campuses and all food retail outlets at each participating university.

As part of the study, we classified foods and beverages according to health, equity and environmental sustainability considerations based on relevant Australian state and territory government guidelines. Due to resource constraints, we did not conduct a comprehensive assessment of the nutritional profile, extent of processing or environmental sustainability of foods and beverages offered within food retail outlets and vending machines. This may have led to misclassification of some products and a less in-depth understanding of the healthiness and environmental sustainability of products available in these settings. Future research could include a more detailed assessment of the nutritional and environmental quality of foods available at universities, in conjunction with the policy and campus audits conducted as part of the Uni-Food tool.

## Conclusion

Universities can play a leading role in creating societal change, including efforts to improve population and planetary health. They have historically been some of the first organisations to support young people’s health, for example, by implementing policies such as ‘smoke-free campuses’ [39]. There is now a major opportunity for universities to lead societal efforts to create healthy, equitable and environmentally sustainable food systems. However, this study showed that the universities assessed lacked comprehensive policies and commitments to improve the healthiness and environmental sustainability of their campus food environments.

Universities in Australia need to take more comprehensive action to improve food environments, including the introduction of strong policies and associated governance structures to address health, equity and sustainability considerations. Actions should encompass multiple food-related settings, including food retail, vending, student accommodation, catering and events. Other stakeholders, including governments, can play a role in incentivising universities to adopt recommended actions.

## Electronic supplementary material

Below is the link to the electronic supplementary material.

Supplementary Material 1

## Data Availability

The data that support the findings of this study are available from the authors on request.
