# Supplementary material for: Benchmarking the healthiness, equity and environmental sustainability of university food environments in Australia, 2021/22
Source: BMC Nutr. 2025 Feb 11;11:38. doi: 10.1186/s40795-025-01029-x (PMC11817424; doi:10.1186/s40795-025-01029-x)
Supplement: Supplementary file 1 — Supplementary Material 1 [file 40795_2025_1029_MOESM1_ESM.docx]

**Supplementary material**

**Figure S1: Example of indicator and related scoring criteria from the University Food Environment Assessment (Uni-Food) tool**

| **Component: University systems and governance** | | |
| --- | --- | --- |
| **Domain: Leadership and planning** | | |
| **Indicator** | | **Scoring criteria** |
| **LSHIP1** | Does the university have a detailed plan or strategy to promote healthy, equitable and environmentally sustainable campus food environments? | 10 points: There is a publicly available, comprehensive and up-to-date plan or commitment outlining measurable and time-bound objectives and strategies to promote healthy, equitable and environmentally sustainable food environments.  7.5 points: There is a publicly available plan or commitment outlining objectives and strategies to promote healthy, equitable and environmentally sustainable food environments, but it is either not comprehensive in the areas covered, or the measures are not specific, measurable or time bound.  5 points: There is a publicly available plan or commitment outlining objectives and strategies to promote the healthiness, equity or environmental sustainability of campuses, but it is not specifically related to food.  2.5 points: No current plan or commitment but work is underway to develop a comprehensive plan outlining objectives and strategies to promote healthy, equitable and environmentally sustainable environments.  0 points: No evidence/No plan or commitment outlining objectives or strategies to promote healthy, equitable and environmentally sustainable campus environments. |

**Supplementary Table S1: Component and domain scores of nine universities using the University Food Environment Assessment tool, 2021/22**

| **University systems and governance** | | | | | | | | | | |
| --- | --- | --- | --- | --- | --- | --- | --- | --- | --- | --- |
| **University** | **Leadership and planning**  **(%)** | **Policies for food retail environments**  **(%)** | **Monitoring and reporting**  **(%)** | | | **Funding and resources**  **(%)** | | | **Stakeholder engagement**  **(%)** | **Component score**  **(%)** ^1^ |
| Monash University | 50 | 60 | 58 | | | 83 | | | 63 | **63** |
| University of Sydney | 95 | 36 | 27 | | | 53 | | | 70 | **48** |
| Deakin University | 85 | 42 | 25 | | | 53 | | | 63 | **48** |
| University of Queensland | 60 | 8 | 40 | | | 65 | | | 93 | **36** |
| University of Tasmania | 63 | 8 | 60 | | | 33 | | | 58 | **29** |
| Flinders University | 63 | 5 | 42 | | | 58 | | | 40 | **28** |
| Western Sydney University | 38 | 2 | 10 | | | 40 | | | 8 | **14** |
| University of Wollongong | 25 | 15 | 0 | | | 8 | | | 5 | **12** |
| Macquarie University | 25 | 0 | 17 | | | 0 | | | 0 | **4** |
| **Median (IQR)** | **60 (25)** | **8 (31)** | **27 (25)** | | | **53 (25)** | | | **58 (55)** | **29 (34)** |
| **Campus facilities and environments** | | | | | | | | | | |
| **University** | **Availability and accessibility**  **(%)** | **Equity**  **(%)** | **Advertising and sponsorship**  **(%)** | **Events and catering**  **(%)** | | | **Personal and community development**  **(%)** | | **Environmental impact**  **(%)** | **Component score (%)** ^1^ |
| Monash University | 97 | 60 | 75 | 83 | | | 96 | | 77 | **80** |
| University of Sydney | 55 | 60 | 65 | 100 | | | 47 | | 82 | **67** |
| University of Queensland | 57 | 57 | 60 | 75 | | | 91 | | 73 | **67** |
| Flinders University | 54 | 48 | 75 | 38 | | | 78 | | 76 | **59** |
| Deakin University | 64 | 47 | 70 | 35 | | | 69 | | 67 | **57** |
| University of Tasmania | 25 | 67 | 85 | 47 | | | 38 | | 80 | **55** |
| University of Wollongong | 63 | 67 | 0 | 25 | | | 72 | | 73 | **55** |
| Macquarie University | 52 | 18 | 50 | 38 | | | 48 | | 57 | **41** |
| Western Sydney University | 39 | 30 | 65 | 5 | | | 20 | | 58 | **34** |
| **Median (IQR)** | **55 (11)** | **57 (13)** | **65 (15)** | **38 (40)** | | | **69 (31)** | | **73 (10)** | **57 (12)** |
| **Food retail outlets** | | | | | | | | | | |
| **University** | **Availability and accessibility**  **(%)** | **Promotion**  **(%)** | **Price**  **(%)** | | **Information**  **(%)** | | | **Environmental impact**  **(%)** | | **Component score**  **(%)** ^1^ |
| University of Tasmania | 52 | 93 | 75 | | 25 | | | 57 | | **61** |
| Deakin University | 51 | 78 | 49 | | 46 | | | 48 | | **53** |
| University of Queensland | 45 | 83 | 58 | | 25 | | | 51 | | **52** |
| University of Wollongong | 52 | 65 | 49 | | 26 | | | 46 | | **48** |
| Flinders University | 44 | 69 | 50 | | 30 | | | 39 | | **46** |
| Monash University | 34 | 47 | 70 | | 30 | | | 37 | | **45** |
| Macquarie University | 47 | 79 | 56 | | 14 | | | 24 | | **45** |
| Western Sydney University | 41 | 56 | 54 | | 20 | | | 32 | | **41** |
| University of Sydney | 40 | 72 | 57 | | 12 | | | 18 | | **40** |
| **Median (IQR)** | **45 (10)** | **72 (14)** | **56 (8)** | | **25 (10)** | | | **39 (16)** | | **46 (7)** |

Notes:

^1^ Component scores (expressed as a %) derived based on domain scores, weighted by domain weightings (refer to Table 1 for domain weightings). Component weightings not applied.
